# Predictors of incompletion of immunization among children residing in the slums of Kathmandu valley, Nepal: a case-control study

**DOI:** 10.1186/s12889-016-3651-3

**Published:** 2016-09-13

**Authors:** Sumina Shrestha, Monika Shrestha, Rajendra Raj Wagle, Gita Bhandari

**Affiliations:** Department of Community Medicine and Public Health, Maharajgunj Medical Campus, Institute of Medicine, Tribhuvan University, Kathmandu, Nepal

**Keywords:** Immunization, Incomplete immunization, Predictors, Slum, Nepal

## Abstract

**Background:**

Immunization is one of the most effective health interventions averting an estimated 2–3 million deaths every year. In Nepal, as in most low-income countries, infants are immunized with standard WHO recommended vaccines. However, 16.4 % of children did not receive complete immunization by 12 months of age in Nepal in 2011. Studies from different parts of the world showed that incomplete immunization is even higher in slums. The objective of this study was to identify the predictors of incompletion of immunization among children aged 12–23 months living in the slums of Kathmandu Valley, Nepal.

**Methods:**

The unmatched case-control study was conducted in 22 randomly selected slums of Kathmandu Valley. The sampling frame was first identified by complete enumeration of entire households of the study area from which 59 incompletely immunized children as cases and 177 completely immunized children as controls were chosen randomly in 1:3 ratio. Data were collected from the primary caretakers of the children. Backward logistic regression with 95 % confidence interval and adjusted odds ratio (AOR) were applied to assess the factors independently associated with incomplete immunization.

**Result:**

Twenty-six percent of the children were incompletely vaccinated. The coverage of BCG vaccine was 95.0 % while it was 80.5 % for measles vaccine. The significant predictors of incomplete immunization were the home delivery of a child, the family residing on rent, a primary caretaker with poor knowledge about the schedule of vaccination and negative perception towards vaccinating a sick child, conflicting priorities, and development of abscess following immunization.

**Conclusion:**

Reduction of abscess formation rate can be a potential way to improve immunization rates. Community health volunteers should increase their follow-up on children born at home and those living in rent. Health institutions and volunteers should be influential in creating awareness about immunization, its schedule, and post-vaccination side effects.

## Background

Globally, 6.3 million children under 5 years of age die each year [1]. An estimated 1.5 million children die annually from diseases that can be prevented by immunization [2]. Unlike many other health interventions, vaccines have both short and long-term benefits not only for individuals but also for the entire population [3]. Following the success of global smallpox eradication program, expanded program on immunization was established in 1974 through World Health Assembly [4]. In most low-income countries including Nepal, infants are immunized with the standard World Health Organization (WHO) recommended vaccines that protect against eight diseases: Bacille Calmette-Guerin (BCG) vaccine which is given once at birth or in first contact to health worker, the measles vaccine given after the completion of 9 months, and the pentavalent (Diphtheria, Pertussis, Tetanus, Hepatitis B, and Haemophilus Influenza B (DPT-HepB-Hib)) vaccine as well as Oral Polio Vaccine (OPV) given three times in 6, 10 and 14 weeks of age [3, 5]. However, 20.6 million infants did not receive measles vaccine through routine immunization services in 2014 and 18.7 million did not complete the 3-dose series of DPT vaccine [6]. The incompletely vaccinated children remain at risk for vaccine-preventable morbidity and mortality [7].

In Nepal, national immunization program has been the high priority program [5]. However, in 2011, for vaccines that should have been completed by 12 months of age, 16.4 % of the children were being left partially immunized by that age. Among children who received BCG vaccine, 14.71 % did not receive measles vaccine. Similarly, dropout between the first and third dose of pentavalent vaccine was 5.2 % [8]. A study done in slums along Bishnumati River in Kathmandu, Nepal in 2008 showed that the overall coverage of BCG, DPT3, OPV3, and measles vaccines were 91.0 %, 88.1 %﻿, 86.6 %, and 67.2 % respectively [9]. Data from neighboring countries like India [10] and Bangladesh [11] showed that immunization coverage is lower in the slums while the proportion of incomplete immunization is higher than that of non-slum settings. Why children in the slums, even when are close to the necessary services, have poor access to immunization is a question that still needs to be explored in the context of Nepal [12]. Thus, the objective of this study was to identify the predictors of incompletion of immunization among children aged 12–23 months residing in the slums of Kathmandu Valley, Nepal.

## Methods

### Study area and design

This study was carried out in Kathmandu Valley where the capital city of Nepal lies. It is a highly populous region of the country having an average population growth almost three times higher than the national data [13]. Difficult access to housing for low-income groups has led informal settlements within the valley. There are 75 slum settlements in the valley [14].

The unmatched case-control study design was chosen to identify independent factors leading to partial immunization during the first year of life. Among 75 slums within the valley, 30 % (22) were chosen randomly using lottery method for data collection to ensure an adequate representation of the study population.

### Study population and sampling

The study population was the children aged 12–23 months. Data were obtained by interviewing a mother or a primary caretaker of each child. A complete enumeration of entire households was done within the selected slums to identify the sampling frame. A child whose family had migrated into the slum when he/she was already 12 months old was excluded from the study. Similarly, those who never received any vaccines were also not included. Cases were identified as children who did not complete recommended vaccination by 12 months of age. Controls were those who received complete vaccines that are supposed to be given within 12 months of age: BCG, OPV, pentavalent and measles vaccines. Altogether 68 incompletely vaccinated children, 187 completely vaccinated and 7 unvaccinated children were identified during complete enumeration. The complete enumeration was conducted from 13 to 30 September 2014 while the final data collection was subsequently started and finished by 8th November 2014.

The sample size was calculated through Epi-Info version 7 [15] considering 80 % power, 95 % confidence interval (CI), the case-control ratio of 1:3, odds ratio 2.5 and proportion of illiterate mothers as 40.57 % among the controls [8]. Among many exposure variables, educational status was chosen since the data for vaccination in relation to educational status was available in Nepalese context. Considering 5 % non-response rate, 240 samples (60 cases and 180 controls) were approached for study. The study participants were chosen randomly from total cases and controls by the use of random number generation technique. Thus, the final analysis included 236 samples (59 cases and 177 controls).

### Data collection tools and techniques

Data were collected by four trained public health graduates using structured questionnaires. A screening questionnaire was used for complete enumeration of entire households to identify the children aged 12–23 months and their immunization status. The information regarding factors related to immunization was taken through a separate questionnaire. Questionnaires were adapted from Nepal Demographic Health Survey (NDHS) 2011 [8], Immunization Coverage Cluster Survey: Reference Manual [16], and through a review of the literature as well as consultation with experts. Questionnaires were first developed in English, then translated into Nepali and back-translated into English. The respondents were the primary caretakers, only those who were directly involved in the immunization of the child. Only two caretakers were fathers while the rest were mothers. The sociodemographic variables, questions related to knowledge, attitude and belief of primary caretakers, and health system factors were incorporated in the questionnaire. The variables that were identified as possible risk factors in previous studies were accounted in this study.

Face to face interview with mother/primary caretaker was done to collect the data. To identify child’s immunization status during complete enumeration, immunization card was observed and, if was not available, primary caretakers were asked to recall vaccines that were given to their child.

### Data processing and analysis

Data were first coded and entered into Epidata version 3.1 [17]. The entered data were then imported into SPSS version 17.0 [18] where data checking, cleaning, data recoding and analysis were performed. In bivariate analysis, chi-square and fisher exact test were performed to check the association between outcome and the carefully dichotomized exposure factors. The exposure variables that showed statistical significance in bivariate analysis were included in the multivariate model after checking multicollinearity. Backward logistic regression was applied to examine the independent association between outcome and exposure variables. The *p*-value < 0.05 was considered to be significant and confidence interval for odds ratio (OR) was set at 95 %.

### Operational definitions

The following operational definitions were used:

- *Primary caretaker*
  Mother or caretaker who had been looking after the child since the first year of life and had been directly involved in vaccinating that child was considered as primary caretaker.
- *Socioeconomic Status*
  Socioeconomic status for the household was assessed by adapting the validated assets from NDHS [9]. Included assets were electricity, radio, television, mobile phone, landline phone, refrigerator, table, chair, bed, sofa, cupboard, computer, fan, clock, dwelling characteristics like roof material, cooking fuel, toilet facility, modern toilet, ownership of home, number of rooms, separate kitchen, and ownership of domestic animals and vehicles. Each household asset was given a factor score generated through principal component analysis. The resulting scores were standardized in relation to standard normal distribution with a mean of zero and a standard deviation of one. Based on scores, households were divided into five socio-economic layers with the same number of individuals in each layer. To check the association with the outcome variable, scores were divided into two categories (Poor and Very Poor).
- *Perceived benefits of vaccination*
  Caretakers who were aware of prevention of specific diseases by immunization were considered to know the benefit of vaccination.
- *Knowledge about vaccine-preventable diseases (VPDs)*
  Respondents who knew four or more VPDs were considered to have good knowledge.
- *Knowledge about the schedule of vaccines*
  The caretakers should know the schedule of BCG, pentavalent3, and measles. Otherwise, they would be considered to have poor knowledge about the schedule of vaccination.
- *Fear of side effects*
  Caretakers who said to have a fear of even one vaccine among BCG, Pentavalent, OPV and Measles were placed in the category of having fear. The child may not be immunized with the vaccine for which the caretaker fears of having side-effects.
- *Perceived efficacy of vaccines*
  It refers to the belief of primary caretaker that the child would not develop a disease against which the vaccination is given. Caretakers who believed in the efficacy of all vaccines were considered to have positive perception. However, if caretakers did not believe the efficacy of even one vaccine, they were considered to have a negative perception. It is because the child would not be immunized with the vaccine for which caretaker does not perceive the efficacy.
- *Perceived adequacy of service providers*
  It refers to a number of vaccine providers in the clinic that the primary caretakers perceived to be adequate.
- *Attitude of service provider*
  The attitude of the service provider was measured on a scale from 3 to 12 through a composite score of three variables. Each variable was measured on 4 points Likert scale.
  - Unpleasant words used by the clinic staff while immunizing the child (Likert scale: Always = 1, Sometimes = 2, Rarely = 3 and Never = 4)
  - Whether the service provider ever explained about the significance of immunization (Likert scale: Never = 1, Rarely = 2, Sometimes = 3 and Always = 4)
  - Whether the staff clearly informed about the date for next vaccination (Likert scale: Never = 1, Rarely = 2, Sometimes = 3 and Always = 4)
  The score was dichotomized at natural break-point: Negative if score 6 and Positive if score > 6.

## Results

From complete enumeration in the study area, 262 children aged 12–23 months were found. Among them, 187 (71.4 ± 0.05 %) received complete vaccination while 68 (26 ± 0.06 %) were incompletely vaccinated. Seven children (2.7 ± 0.02 %) had not received any of the vaccines. Regarding specific vaccines, 249 (95.0 %) children received BCG, 216 children (84.0 %) received the three doses of OPV, 223 children (85.1 %) received the three doses of pentavalent and 211 (80.5 %) received measles vaccine. One child (0.4 %) received BCG only. Similarly, 247 children (94.3 %) were given BCG + Penta1, 238 (90.8 %) BCG + Penta2 and 219 (83.6 %) BCG + Penta3. Likewise, 252 children (96.2 %) were immunized with OPV1 + Penta1, 240 (91.6 %) with OPV2 + Penta2, and 216 (82.4 %) with OPV3 + Penta3. Five children (1.9 %) were given Penta1 (no BCG), 4 (1.5 %) given Penta2 (no BCG), and 4 (1.5 %) given Penta3 (no BCG). BCG + Penta3 (no measles) were given to 25 children (9.5 %) while Penta3 + Measles (no BCG) were given to 4 children (1.5 %). Among the partially immunized children, the dropout rate from pentavalent first to third was 44.6 % and BCG to measles vaccine was 61.3 %. The vaccination card was the evidence of immunization among 43.6 % of the total children. Among partially immunized children, 44.1 % had vaccination card.

As per the study design, 236 (59 cases and 177 controls) randomly selected children were included in the analysis. The proportion of female children was higher in the case than in control group. Both cases and controls had a higher percentage of first-born children than the subsequent-born children. The proportion of children delivered at home was much higher in cases. The mean age of caretakers of cases was 23.8 ± 4.9 years while it was 25.2 ± 4.4 years for the control group. More than one-third of the respondents were a housewife in both groups, but the proportion of caretakers involved in daily-wage work was higher among the cases. The proportion of cases decreased with increasing educational level but the proportion of controls was high among educated caretakers. Among ethnic groups, the proportion of Madhesi, Dalit, and religious minorities were higher in cases than in controls. The proportion of cases was higher in children with nuclear family, those living in rent, and from a family with the lowest socioeconomic condition (Table 1).

**Table 1:** Sociodemographic characteristics

Variables	Case (%) (*n* = 59)	Control (%) (*n* = 177)
Gender^#^		
Male	33 (55.9)	107 (60.5)
Female	26 (44.1)	70 (39.5)
Birth order^#^		
First	26 (44.1)	100 (56.5)
Second	20 (33.9)	57 (32.2)
Third or above	13 (22.0)	20 (11.3)
Birth Place^#^		
Semi/Governmental Institution	34 (57.6)	132 (74.6)
Home	19 (32.2)	21 (11.9)
Private Institution	6 (10.2)	24 (13.6)
Age ^Φ^		
<20	12 (20.3)	14 (7.9)
20–24	22 (37.3)	75 (42.4)
25–29	17 (28.8)	47 (26.6)
≥30	8 (13.6)	41 (23.2)
Ethnicity		
Janajati	22 (37.3)	108 (61.0)
Upper caste	10 (16.9)	39 (22.0)
Madhesi	16 (27.1)	14 (07.9)
Dalit	6 (10.2)	16 (09.0)
Religious Minorities	5 (08.5)	0 (0)
Occupation ^Φ^		
Housewife	46 (78.0)	137 (77.4)
Daily wage/ Labour	8 (13.5)	16 (09.0)
Others	5 (08.5)	24 (13.5)
Education level ^Φ^		
Illiterate	22 (37.3)	26 (14.7)
Literate/ Primary	22 (37.3)	64 (36.2)
Secondary	14 (23.7)	68 (38.4)
SLC and above	1 (01.7)	19 (10.7)
Type of family		
Nuclear	40 (67.8)	103 (58.2)
Joint	17 (28.8)	73 (41.2)
Extended	2 (03.4)	1 (00.6)
Type of residence		
Own	20 (33.9)	123 (69.5)
Rent	39 (66.1)	54 (30.5)
Socioeconomic status		
Lowest	24 (40.7)	24 (13.6)
Second	15 (25.4)	31 (17.5)
Middle	8 (13.6)	40 (22.6)
Fourth	8 (13.6)	39 (22.0)
Highest	4 (06.8)	43 (24.3)

# = variable related to child, Φ = variable related to primary care-taker

### Association between outcome and exposure variables

In bivariate analysis, thirteen variables were found to be statistically associated with immunization status of the children. A child born third or above in order was twice likely to receive partial immunization than the former. Children delivered at home were highly likely to incomplete the immunization schedule (OR = 3.53, CI: 1.73–7.19) in comparison to those delivered at a health institution. Similarly, children having caretaker below 20 years of age were more at risk to receive partial immunization as compared to those having caretaker aged 20 years or more. Illiterate caretakers were more likely to partially immunize their children than literate caretakers. Children from Dalit/ Madhesi/ religious minorities were four times likely to be the case as compared to Janajati/ upper caste child. Children born to poorer families were highly at risk of being incompletely immunized than those born to families with the better economic condition. Type of residence also had a significant association with immunization status of the child. No significant difference in risk was found between male and female children, children having employed and unemployed caretakers, and those living in a nuclear family and joint/extended family. The association between sex, birth order and immunization status were also analyzed. However, the statistical association was not found between immunization status of the female child and her birth order, and the male child and his birth order. Similarly, statistical significance was also not observed between sex and immunization status among former-born children as well as sex and immunization status among subsequent-born children (Table 2).

**Table 2:** Association between sociodemographic characteristics and immunization status of children

Variables	Case (%) (*n* = 59)	Control (%) (*n* = 177)	*p*-value	Crude OR for complete immunization (95 % CI)
Gender^#^				
Male (vs Female)	33 (55.9)	107 (60.5)	0.541	1.20 (0.66–2.19)
Birth order^#^				
First/ Second (vs ≥ third)	46 (78.0)	157 (88.7)	0.039*	2.22 (1.02–4.80)
Birth Place^#^				
Health Institution (vs Home)	40 (67.8)	156 (88.1)	<0.001*	3.53 (1.73–7.19)
Age^Φ^				
≥20 (vs <20)	47 (79.7)	163 (92.1)	0.008*	2.97 (1.29–6.862)
Employment status^Φ^				
Employed (vs Unemployed)	13 (22.0)	40 (22.6)	0.928	1.03 (0.51–2.10)
Educational status^Φ^				
Literate (vs Illiterate)	37 (62.7)	151 (85.3)	<0.001*	3.45 (1.76–) 6.76
Ethnicity				
Janajati/Upper caste (vs other^┼^)	32 (54.2)	147 (83.1)	<0.001*	4.13 (2.17–7.88)
Type of family				
Nuclear (vs Joint/Extended)	40 (67.8)	103 (58.2)	0.193	0.66 (0.35–1.23)
Type of resident				
Own (vs Rent)	20 (33.9)	123 (69.5)	<0.001*	4.44 (2.37–8.31)
Socioeconomic status				
Poor (vs Very Poor)	16 (27.1)	102 (57.6)	<0.001*	3.66 (1.91–6.98)

* = *p*-value <0.05

# = variable related to child, Φ = variable related to primary care-takers ┼ = Dalit/ Madhesi/ Religious minorities

Primary caretaker’s knowledge about benefits and schedule of vaccines had a significant statistical association with incomplete immunization status of the child. Conflicting priorities during vaccination day were less likely to lead to the completion of vaccination. The perception that vaccine should not be given to the sick child was also significantly associated with incompletion of immunization during bivariate analysis (Table 3).

**Table 3:** Association between primary care-takers’ knowledge, attitude and belief and immunization status of children

Variables	Case (%) (*n* = 59)	Control (%) (*n* = 177)	*p*-value	Crude OR for complete immunization (95 % CI)
Knowledge on benefit of vaccine				
Yes (vs No)	52 (88.1)	173 (97.7)	0.006^*^	5.82 (1.64–20.67)
Knowledge on VPDs				
Good (vs Poor)	3 (05.1)	25 (14.1)	0.063	3.07 (0.89–10.57)
Knowledge on schedule of vaccines				
Good (vs Poor)	9 (15.3)	79 (44.6)	<0.001^*^	4.48 (2.08–9.66)
Conflicting Priorities				
Yes (vs No)	13 (22.0)	13 (7.3)	0.002^*^	0.28 (0.12–0.65)
Fear of side effects				
Yes (vs No)	16 (27.1)	38 (21.5)	0.372	0.73 (0.37–1.45)
Perceived Efficacy of vaccine				
Positive (vs Negative)	48 (81.4)	139 (78.5)	0.643	0.84 (0.40–1.77)
Perception of vaccinating the sick child				
Positive (vs Negative)	9 (15.3)	59 (33.3)	0.01^*^	2.78 (1.28–6.03)

* = *p*-value <0.05

Table 4 shows that developing abscess after immunization and severe pain at the injection site were statistically associated with incomplete immunization status of the child. Development of fever following immunization was not found statistically significant. Convenience of opening hour, extent of waiting, stock out of vaccines as well as attitude and adequacy of service provider did not show statistical association with incomplete immunization status of the child (Table 4).

**Table 4:** Association between health system factors and immunization status of children

Variables	Case (%) (*n* = 59)	Control (%) (*n* = 177)	*p*-value	Crude OR for complete immunization (95 % CI)
Convenient opening hour				
Yes (vs No)	54 (91.5)	163 (92.1	0.890	1.08 (0.37–3.132)
Perceived extent of waiting				
Reasonable (vs Not reasonable)	51 (86.4)	150 (84.7)	0.751	0.87 (0.37–2.04)
Abscess after immunization				
Yes (vs No)	14 (23.7)	12 (06.8)	<0.001^*^	0.23 (0.10–0.54)
Severe pain in injection area				
Yes (vs No)	11 (18.6)	15 (08.5)	0.031^*^	0.40 (0.17–0.94)
Fever after immunization				
Yes (vs No)	37 (62.7)	90 (50.8)	0.113	0.61 (0.34–1.13)
Stock out of vaccines				
Yes (vs No)	5 (08.5)	13 (7.3)	0.777	0.86 (0.29–2.51)
Attitude of service provider				
Positive (vs Negative)	53 (92.8)	166 (93.8)	0.382	1.71 (0.60–4.84)
Perceived adequacy of service provider				
Yes (vs No)	49 (83.1))	148 (83.6)	0.919	1.04 (0.47–2.29)

* = *p*-value <0.05

### Independent predictors of incompletion of immunization

In the final logistic regression model, six variables (the birthplace of a child, type of residence, caretaker’s knowledge about the schedule of vaccination, conflicting priorities, perception of vaccinating a sick child, and development of abscess following immunization) were found to be independent predictors of incomplete immunization. Children delivered at home were more likely to remain incompletely vaccinated than those born at health institution (OR: 2.78, 95 % CI: 1.14–6.80). Children living in rented home had a higher risk of being incompletely vaccinated than those living on their own (OR: 3.77, 95 % CI: 1.70–8.38). Primary caretakers with poor knowledge about the schedule of vaccination were more likely to incomplete the recommended vaccination of children (OR: 3.90, 95 % CI: 1.60–9.51). Primary caretakers who had conflicting priorities during the days of vaccination were less likely to complete vaccination of their children (OR: 0.22, 95 % CI: 0.08–0.61). Similarly, caretakers with negative perception towards vaccinating sick child were three times likely to have partially immunized children than those with a positive attitude (OR: 3.23, 95 % CI: 1.20–8.73). Development of abscess in the vaccination site of a child was less likely to be a factor for incomplete immunization of the child (OR: 0.14, 95 % CI: 0.05–0.39) (Table 5).

**Table 5:** Independent predictors of immunization status of the children

Variables	*p*-value	Adjusted OR for complete immunization (95 % CI)
Place of birth of child		
Health Institution (vs Home)	0.025*	2.78 (1.14–6.80)
Type of residence		
Own (vs Rent)	0.001*	3.77 (1.70–8.38)
Knowledge on schedule of immunization		
Good (vs Poor)	0.003*	3.90 (1.60–9.51)
Conflicting priorities		
Yes (vs No)	0.004*	0.22 (0.08–0.61)
Perception of vaccinating the sick child		
Positive (vs Negative)	0.021*	3.23 (1.20–8.73)
Abscess after immunization		
Yes (vs No)	<0.001*	0.14 (0.05–0.39)

* = *p*-value <0.05

We found the differences in the risk factors between cases and controls that did and did not have the immunization card. For those having a card, age, birth order, type of residence, knowledge about the schedule of immunization, and perception towards vaccinating a sick child were found as the risk factors for incomplete immunization of the child. Among those who did not have the card, birthplace, conflicting priorities, and development of abscess were found to be the risk factors.

## Discussion

The study found that 26 % of slum children aged 12–23 months had not completed the recommended schedule of immunization. It is higher than the national data 16.4 % [8]. This finding is also consistent with the studies from different states of India which show that 30 % of the slum children have partial immunization [19, 20]. Like in studies carried out in other slums [20–23], this study also found that majority of the partially immunized children were not immunized with measles vaccine and third dose of pentavalent vaccines. The major reason might be the development of an abscess in the vaccination site which is also one of the predictors of incomplete immunization identified in this study. The caretaker may not want to continue vaccination of the child after experiencing the abscess in the previous episode. Review of gray literature [24, 25] also showed that unpleasant experiences at health institution including post-vaccination abscess are associated with non-vaccination and under-vaccination. It is a serious problem in the health system and thus reducing the abscess formation can be a potential way to improve immunization rates.

Factors determining incompletion of immunization are complex. Among sociodemographic factors, place of birth and type of residence were found to be independent factors that lead to incomplete immunization status of the child. Similar to our study, home delivery was found to be a risk factor in case-controlstudies carried out in Ethiopia [26, 27]. The studies in a similar circumstance in India [21] and among migrant children in China [28] also supported the finding. It might be because the first dose of vaccination is given just after birth in health institution from which the mother or caretaker gets suggestions of when to come to receive other vaccines. Similarly, findings showed that children living in the rented house were nearly four times likely to be partially immunized than those living in their own house. Study on factors associated with BCG versus Measles dropout in Nepal also showed greater dropout rate among those living in rented house [29]. As a frequent change of rent occurs in the slum area, it might be difficult to find the place of vaccination for the caretakers when shifted from one slum to another. The problem might be more prominent among those who changed their place of living before completion of the immunization schedule of the child. Sex of the child was found as an important determinant of childhood immunization by many studies [19, 29–31]. However, our findings showed no association. This might be because of more awareness and advocacy about gender equality in the country to reduce the social differences between male and female.

Children whose caretakers did not know about the schedule of vaccination were more likely to remain partially immunized. This finding is similar to the descriptive study carried out in India [21]. A recent case-controlstudy carried out in Ethiopia [26] also showed that maternal immunization-related knowledge was one of the significant predictors of immunization defaulting. The possible explanation could be that if the caretakers have the knowledge about the schedule of the vaccination, they immunize the children on time. Ignorance on dates to immunize the child might also account for failure to complete recommended vaccines.

Conflicting priority was found to be another independent predictor of incomplete immunization. Review of gray literature for non-vaccination [24, 25] has also shown conflicting priorities to be the risk factor in most of the studies including those in neighboring countries like India and Bangladesh. Conflicting priorities like the need to take care of sick or other children, caretakers’ own illness, weddings, and funerals as well as cultural feast/ festivals might be the major obstacles in our scenario. Similarly, findings of our study showed that the belief that sick child should not be vaccinated is one of the reasons for the child to be incompletely immunized. This perception was associated with dropout in other studies carried out in Nepal [29] and slums of India [20, 32]. The parental perception that their child is too sick to vaccinate had also led to under-vaccination [24]. A qualitative part of the mixed study in Istanbul has mentioned that mothers were afraid of injecting vaccines to sick children thinking that they would suffer more [33]. It might also lead to postponing the date of vaccination, which was an important predictor of incomplete immunization in a case-controlstudy carried out in Ethiopia [34].

Studies have shown that fear of side effects was found as an important factor leading to under-immunization [25, 35–37]. A review of the gray literature [24] has also shown that perceived efficacy is one of the major reasons behind under-vaccination. However, our study did not show any statistical association of these variables with incomplete immunization.

Other variables related to health system like convenient opening hour, perceived length of waiting time, and stock out of vaccines showed no association in this study. However, these variables were shown as the reasons related to under-vaccination or non-vaccination in a systematic review of the literature [24, 25, 35]. Similarly, perceived inadequacy of service providers was statistically insignificant in our study but was found as an important factor leading to partial immunization in a review study [35]. Service providers’ attitude was also an important reason for under-vaccination in the review of gray literature carried out by Favin et al. [24] but not in our study.

In this study, variables relevant in terms of slum area were assessed to determine the independent predictors of incomplete immunization of slum-children using a community-based case-controlstudy. However, recall bias was more likely as the immunization status of the children was relied on primary caretakers’ recall for those children who did not have immunization card. To minimize it, separate questions for each age-appropriate vaccine, careful probing and cross-questioning were done.

## Conclusion

This study provides deeper insights into the independent factors that lead to incomplete immunization of the children living in slums. This is expected to be a guideline for policymakers as national immunization program has been the high priority program in Nepal with more focus on a rural area as well as urban poor. Based on the findings, reducing the rate of abscess development can be a potential way to increase the immunization completion rate. Similarly, post-vaccination side effects and their management should be clearly explained to the primary caretakers during immunization. Health volunteers, appointed for immunization in the slums, should focus more on those children delivered at home and those living in rent within the slum. Health institutions providing immunization services need to educate mothers and caretakers about the schedule of immunization so that they would be aware of when to come next to immunize their children.

## Abbreviations

BCG: Bacille Calmette-Guerin
DPT: Diphtheria, Pertussis, Tetanus
NDHS: Nepal Demographic Health Survey
OPV: Oral polio vaccine
OR: Odds ratio
Penta: Pentavalent (Diphtheria, Pertussis, Tetanus, Hepatitis B, Haemophilus Influenza B)
VPD: Vaccine preventable disease
vs: Versus
WHO: World Health Organization
